# Choosing words wisely: Language, metaphor, and psychological challenges after pulmonary embolism

**DOI:** 10.1002/hem3.70232

**Published:** 2025-10-28

**Authors:** Gerard Gurumurthy, Jecko Thachil, Kerstin de Wit, Stephen P. Hibbs

**Affiliations:** ^1^ The Queen Elizabeth Hospital Kings Lynn NHS Foundation Trust Kings Lynn United Kingdom; ^2^ Manchester Academic Health Science Centre The University of Manchester Manchester United Kingdom; ^3^ Division of Emergency Medicine, Department of Medicine McMaster University Hamilton Ontario Canada; ^4^ Department of Emergency Medicine Queen's University, Kingston Ontario Canada; ^5^ Wolfson Institute of Population Health Queen Mary University of London London United Kingdom

Traditionally, clinical follow‐up after pulmonary embolism (PE) has focused on medical therapy: the length of time a patient is anticoagulated, type and dose of anticoagulant, and mitigation of bleeding risk. There is often limited time to address other factors in a patient's recovery and wellbeing. However, when asked, patients describe a negative emotional burden which can persist years after the diagnosis, even when their management and physical recovery has been uncomplicated.^1^, ^2^ Fear, anxiety, and hypervigilance are faced by many PE patients but are often unexplored in clinical encounters.^2^ The choice of language used by healthcare professionals can inflame these psychological challenges or create space for them to be articulated and better understood. Here, we explore how clinician language can fail to address psychological consequences of PE and even exacerbate negative mental health.

PE survivors frequently endure significant psychological challenges. Studies suggest that roughly 3% of patients meet the criteria for posttraumatic stress disorder at follow‐up.^2^ A further 21.3% experience clinically significant anxiety and 18.3% report depressive symptoms within 3 months of their event.^3^ Nearly one in five continue to endure anxiety or depression up to 2 years post‐PE.^4^ This high prevalence of psychological morbidity correlates with a reduction in wider health‐related quality‐of‐life. The mean SF‐36 Mental Component Summary score in a cohort of 251 PE survivors was 43.6 ± 19.8 compared to a normative mean of 50.^5^ It showed persistent impairments in social functioning, vitality and role‐emotional domains relative to age‐ and sex‐matched population norms.^5^

## CLINICIAN LANGUAGE AT THE POINT OF DIAGNOSIS

PE can induce a sense of fear and anxiety among healthcare staff. This is especially common when the condition is first recognised. This anxiety may be transferred to patients through a number of ways.^6^ Sudden, simultaneous attention from a succession of doctors and nurses signals a more serious condition. The sense of fear can be exacerbated when no single healthcare provider sits down with the patient in a quiet environment, calmly explains what the diagnosis is, provides written information and answers questions.^7^ Instead, some healthcare providers use terms that patients do not understand (e.g., ‘pulmonary embolism’), and may inadvertently transfer a disproportionate sense of fear and emergency by their nonverbal behaviour. Language and clinician behaviour become salient memories and shape a patient's perception of their health. To appreciate the power of metaphors used by medical staff, consider these recollections from PE patients:

*‘[The doctor] told me after, when I was leaving [the hospital], “a lot of people don't get through this” […] I was one of the lucky ones. […] As the one doctor said, “this is a rare opportunity to talk to someone who made it through.” (laughs) (69‐year‐old man)’* ^2^

*‘When [the doctor] said it was a blood clot, […] and it could go any minute, I could have a stroke, I could have a heart attack… […] that was a whole other ball game. […] That has got to be one of the most traumatic things in my life. (63‐year‐old woman)’* ^2^

An individual in the same study recalled phrases from clinicians such as ‘you're one of the lucky ones’ as a source of ongoing hypervigilance for recurrent thrombosis or other complications. It is quite possible that no clinician used these exact words at the point of diagnosis, yet these quotes exemplify the message the patient hears (and remembers). A scoping review found that most clinicians provide only brief discharge instructions, often in technical language, leaving most patients with no clear next steps.^8^ For healthcare providers, perhaps the most important skill is to demonstrate a PE diagnosis is not a rare occurrence, that the healthcare team are skilled in dealing with this routine diagnosis and prioritise immediate patient education in an appropriate, quiet environment.

## IMPROVING MENTAL HEALTH OUTCOMES IN CLINIC

Long after PE treatment has been initiated and the acute thrombus has resolved, patients are predisposed to worry that the clot may get worse, move or recur. Because some clinics have focused only on medical treatment, patients can feel abandoned by a ‘fast‐track’ system with no clear next steps for questions or support.^2^ Below, we share responses from participants in two different studies that illustrate the psychological challenges individuals face following diagnosis with a PE:

*‘Oh, if I get an ache, my one leg if I get a bit of an ache in that it's like ‘oh my god, there's something happening there again’* ^1^

*‘Because it's so similar to the symptoms… as a PE that's the problem. Chest pains, can't breathe, heart racing. I don't think I will ever not be frightened of them cos no matter how much I read into them you could always have that [PE]. There's a very small chance it could happen and you should never ignore)’* ^1^

*‘In the beginning I was very very very weary, very scared. I would only have to get a sort of sign or niggle in my chest and I would start to panic thinking that it was going to happen again or I'd end up in a heap on the floor’* ^9^

Clinic pathways may provide little space to address such challenges. All PE patients should be counselled to expect these natural concerns about symptoms, and given instructions for how to handle the anxiety, when to seek emergency care and when to turn to relaxation and reassurance techniques. Such fears of causing recurrence can also create reluctance to engage in physical exercise.^10^ Patients should be provided with standard advice on returning to normal activities, including exercise.

Psychological consequences of PE may not be readily disclosed in follow‐up clinics. Across several studies, patients routinely explained that they considered feelings of anxiety or depression ‘outside the scope’ of their thrombosis management and were unsure whom to contact, particularly during the interval between discharge and their first thrombosis‐clinic appointment.^2^, ^11^, ^12^ Negative mental health should be routinely screened for in clinic, for example, by the use of validated tests such as the PHQ‐9 and GAD‐7 questionnaires.^13^ Psychological distress can be managed by repeat review of what PE is (i.e., not a disease associated with stroke or heart attack, a time‐limited event which is managed with medications), directly addressing a patient's fears/concerns, offering access to support groups, and arranging specialist counselling. Routine integration of mental well‐being screening in follow‐up has been shown to improve patient satisfaction.^14^

Thoughtful clinical approaches may be able to mitigate the lingering fear and confusion that can follow a PE diagnosis. For instance, Klok et al. recently described the creation of an interactive, evidence‐based ‘provider tool‐kit’ that maps the VTE patient journey into immediate, intermediate, and long‐term phases.^15^ The tool‐kit offers concrete examples of ‘good language’ scripts, visual aids, and talking points tailored to each timepoint:

- Begin by gauging baseline knowledge: ‘*What do you know about a blood clot?*’.
- Then, confirm understanding via teach‐back: *‘Can you please tell me in your own words what I just told you? This will ensure that I provided you all the right information*’.
- Normalise emotions without alarmism: ‘*It's perfectly normal to feel worried about this, but if you're not that's fine too. If you are worried, then here's some things you can do*’.
- Deliberately avoid phrases like ‘*you dodged a bullet’*, which can worsen anxiety.^15^

To counter feelings of abandonment and uncertainty, Mishra et al. recommend providing every patient with a one‐page ‘PE care plan’ that clearly lists any follow‐up imaging dates, a direct helpline for anticoagulation questions, and peer‐support contacts where available, to reinforce continuity of care and reassure patients that someone remains responsible for their recovery.^8^

Fear and concern after PE is a rational response to a significant event with long‐term consequences. However, the choice of language used by clinicians can inflame or alleviate psychological distress. Best practice involves clear post‐discharge lines of communication, and language which normalises unpleasant emotions and avoids frightening metaphors. As imaging protocols and anticoagulant algorithms are refined, clinicians should remember the first therapeutic intervention they employ: **language**

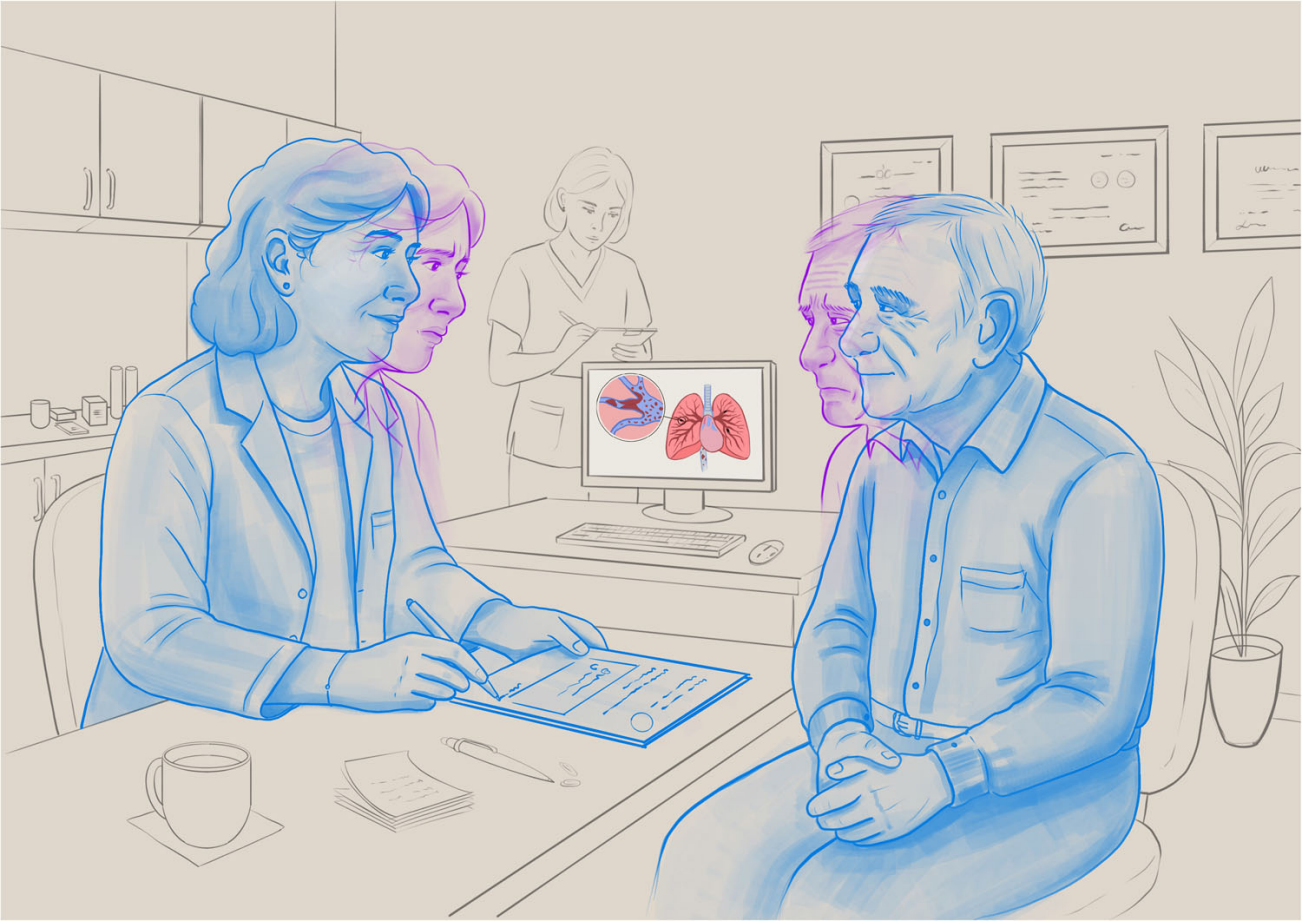

.

## AUTHOR CONTRIBUTIONS

**Gerard Gurumurthy**: Conceptualization; writing—original draft; project administration; writing—review and editing. **Jecko Thachil**: Writing—review and editing. **Kerstin de Wit**: Writing—review and editing. **Stephen P. Hibbs:** Conceptualization; writing—original draft; project administration; writing—review and editing.

## CONFLICT OF INTEREST STATEMENT

The authors declare no conflict of interest.

## FUNDING

SPH is supported by a HARP doctoral research fellowship, funded by the Wellcome Trust (Grant number 223500/Z/21/Z).

## Data Availability

Data sharing not applicable to this article as no datasets were generated or analysed during the current study.
